# Temporal correlation between malaria and rainfall in Sri Lanka

**DOI:** 10.1186/1475-2875-7-77

**Published:** 2008-05-06

**Authors:** Olivier JT Briët, Penelope Vounatsou, Dissanayake M Gunawardena, Gawrie NL Galappaththy, Priyanie H Amerasinghe

**Affiliations:** 1 International Water Management Institute, PO Box 2075, Colombo, Sri Lanka; 2 Swiss Tropical Institute, Socinstrasse 57, PO Box CH-4002, Basel, Switzerland; 3 US Agency for International Development, PO Box 7856, Kampala, Uganda; 4 Anti Malaria Campaign, Head Office Colombo, Sri Lanka; 5 International Water Management Institute Sub Regional Office for South Asia, c/o ICRISAT, Patancheru, AP 502 324, Andhra Pradesh, India

## Abstract

**Background:**

Rainfall data have potential use for malaria prediction. However, the relationship between rainfall and the number of malaria cases is indirect and complex.

**Methods:**

The statistical relationships between monthly malaria case count data series and monthly mean rainfall series (extracted from interpolated station data) over the period 1972 – 2005 in districts in Sri Lanka was explored in four analyses: cross-correlation; cross-correlation with pre-whitening; inter-annual; and seasonal inter-annual regression.

**Results:**

For most districts, strong positive correlations were found for malaria time series lagging zero to three months behind rainfall, and negative correlations were found for malaria time series lagging four to nine months behind rainfall. However, analysis with pre-whitening showed that most of these correlations were spurious. Only for a few districts, weak positive (at lags zero and one) or weak negative (at lags two to six) correlations were found in pre-whitened series. Inter-annual analysis showed strong negative correlations between malaria and rainfall for a group of districts in the centre-west of the country. Seasonal inter-annual analysis showed that the effect of rainfall on malaria varied according to the season and geography.

**Conclusion:**

Seasonally varying effects of rainfall on malaria case counts may explain weak overall cross-correlations found in pre-whitened series, and should be taken into account in malaria predictive models making use of rainfall as a covariate.

## Background

Malaria is a complex disease and its transmission and prevalence is influenced by many factors, amongst which (variability in) climatic conditions are considered to play a major role. With increasing weather variability and ability to forecast weather, there is an interest in developing systems for malaria forecasting that incorporate weather related factors as explanatory variables. Many studies in various parts of the world have linked malaria time series to weather variables such as rainfall, temperature and humidity. For instance, by using polynomial distributed lag models, Teklehaimanot and colleagues [1] found that malaria was associated with rainfall and minimum temperature (with the strength of the association varying with altitude) in Ethiopia. Worrall and colleagues [2] used rainfall and maximum temperature at a lag of four months to successfully fit a biological transmission model to malaria case data in a district in Zimbabwe. Craig and colleagues [3] linked inter-annual differences in malaria to rainfall and temperature in South Africa. Sri Lanka has a long history of researching the links between rainfall and malaria and many studies observed links between the two [4-7]. Yet others did not find a strong [8] or an obvious correlation [9]. A study in Sri Lanka incorporating rainfall as a linear or non linear explanatory variable into a (seasonal) auto-regressive integrated moving average (ARIMA) model showed little improvement in malaria prediction over ARIMA models without a rainfall predictor [10].

**Figure 1:**
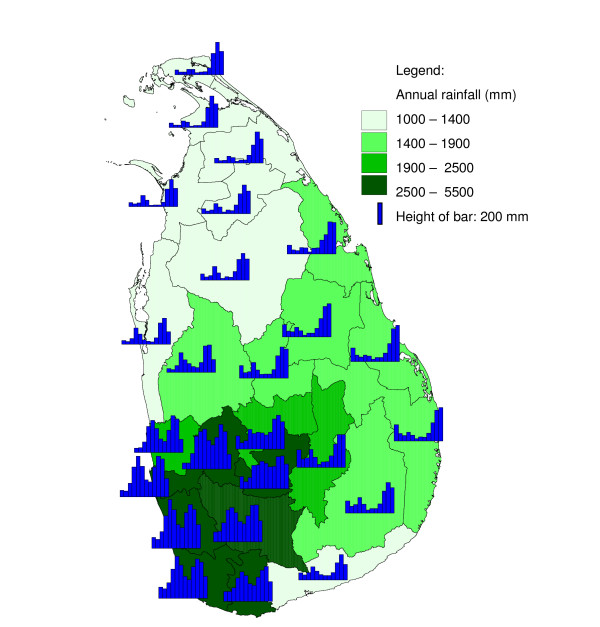
**Geometric mean seasonality and annual geometric mean total of rainfall**. Geometric mean monthly rainfall from January (bar on far left) to December (bar on far right), calculated over the period January 1971 to December 2005, and the total geometric mean annual rainfall in districts of Sri Lanka. The height of the bar in the legend represents 200 mm. The classification of the annual rainfall follows the common delineation into a wet zone (>2500 mm per annum), intermediate zone, and a dry (<1900 mm per annum) zone, with a "very dry" category for rainfall <1400 mm per annum.

**Figure 2:**
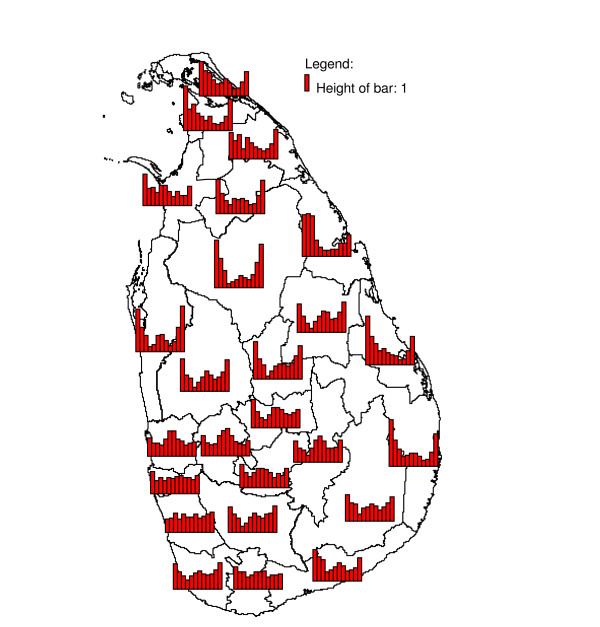
**Geometric mean seasonality of detrended malaria cases**. Geometric mean monthly number of malaria cases from January (bar on far left) to December (bar on far right), calculated over the period January 1972 to December 2005, after detrending, in districts of Sri Lanka. The height of the bar in the legend represents 1, (because of detrending, no unit is given).

Weather affects the malaria incidence mostly through its effects on both the mosquito vector (species, population dynamics, gonotrophic cycle and survivorship [11]) and the development of the malaria parasite inside the mosquito vector. In Sri Lanka, the main malaria vector *Anopheles culicifacies *breeds primarily in river bed pools [12] which occur during dry periods, but also in other breeding sites such as seepage areas next to irrigation tanks, hoof prints, and abandoned gem mining pits. The spatial variation in annual precipitation (Figure 1) has been linked to spatial variation in malaria endemicity in Sri Lanka [13] by early malariologists who used the concept of a classification of the country into a wet, intermediate and dry zone [6] based on the amount of rainfall received during the south-west monsoon. The region receiving the most annual precipitation has the least malaria, and endemicity increases with decreasing annual rainfall. The fact that the districts in the extreme south west of the island (Galle and Kalutara) have always been virtually free of malaria is attributed to the wet climate in which rivers flow year round without pooling. In the south west, only a drought might cause pooling in rivers and hence create conditions suitable for the breeding of *An. culicifacies*. For example, districts with wet and intermediate annual rainfall in this region have repeatedly been affected by malaria epidemics, mostly attributed to droughts due to a failing south-west monsoon (which occurs normally between February and July), while districts towards the north and east with dryer climates (and with a higher malaria endemicity) were less affected [6]. Hence a negative correlation between rainfall and malaria is expected in districts in the wet and intermediate rainfall zones. In contrast, towards the north and east, where the climate is much dryer (particularly during April – September) and rivers often run dry, rainfall creates new puddles, especially following a period of drought. The relation between rainfall and malaria may not only change over space, but also depending on the season. Both rainfall and malaria show a marked seasonality which is bimodal in the south west and increasingly monomodal towards the north and east [14] (Figure 1 and Figure 2). There is a very interesting duality in the relationship of rainfall with malaria. Although malaria prevalence (not shown in this paper, see *e.g. *[13]) decreases with annual rainfall in space, study of the seasonality of both (Figure 1 and Figure 2) reveals that malaria follows rainfall with a few months delay, a low seasonal rainfall peak being followed by a low seasonal malaria peak, and a high seasonal rainfall peak being followed by a high seasonal malaria peak. In the present paper, the relationship between rainfall and malaria incidence in Sri Lanka was investigated allowing for spatial variability in the relationship using (i) cross-correlation analysis, (ii) cross-correlation analysis with pre-whitening (prior removal of seasonality and auto-correlation in the series), (iii) inter-annual analysis and (iv) seasonal inter-annual analysis allowing for seasonal variability in the relationship. Only the first of these four approaches has been used to study malaria and rainfall relationships in Sri Lanka previously, and only in limited geographic areas. A better understanding of the relationship between rainfall and malaria may help to improve forecasting of changes in malaria incidence.

## Methods

### Data

Since January 1972, the Sri Lankan national malaria control programme, the Anti Malaria Campaign (AMC), has collected monthly confirmed malaria case data from health facilities aggregated by medical officer of health (MOH) areas (which represent sub district health administrative divisions). This data up to December 2005 was cleaned and aggregated to district resolution. For each district, for each month, the mean rainfall was extracted from monthly rainfall surfaces for the period January 1971 – December 2005. Both rainfall and malaria datasets are described in detail elsewhere [10].

### Statistical analysis

The relationship between rainfall and malaria incidence was investigated using (i) cross-correlation analysis, (ii) cross-correlation analysis with pre-whitening, (iii) inter-annual analysis and (iv) seasonal inter-annual analysis allowing for temporal variability in the effect.

### Cross-correlation analysis

Cross-correlations between detrended monthly malaria case count time series and monthly total rainfall [8] were analysed to detect the time lag(s) of rainfall preceding malaria at which the series show strongest correlation.

**Figure 3:**
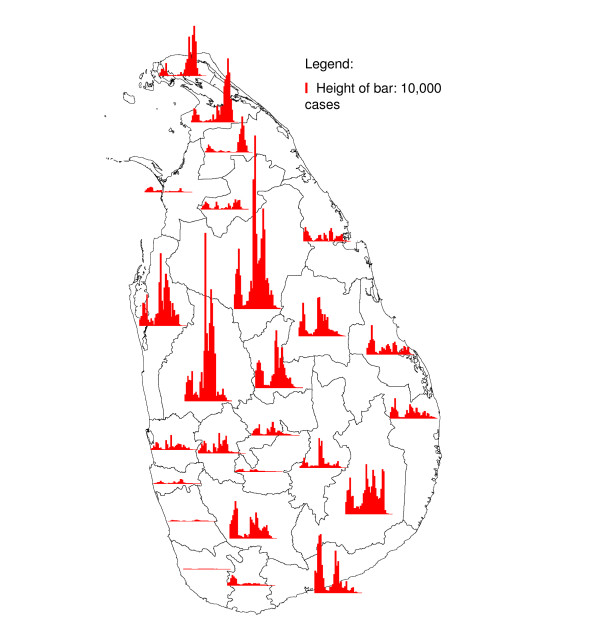
**Annual malaria cases**. Annual number of malaria positive cases from 1972 (bar on far right) to 2006 (bar on for left) in districts in Sri Lanka. The height of the bar in the legend represents 10,000 cases.

Malaria time series showed strong long term fluctuations for most districts in Sri Lanka (Figure 3). However, in rainfall time series these long term fluctuations were absent. Therefore, it was expected that rainfall could not explain the long term fluctuations in malaria, which were probably related to other factors, such as malaria control and population changes. The long term fluctuations masked the correlation between malaria and rainfall and since no information on the underlying factors was available in the data, the long term fluctuations needed to be removed prior to calculating cross-correlations. It was assumed that monthly malaria case count data *y*_*t*_, after the transformation *y*'_*t *_= log(*y*_*t *_+ 1) follow a seasonal model [15] of the form:

*y*'_*t *_= *m*_*t *_+ *S*_*t *_+ *ε*_*t*_

where *m*_*t *_is the mean level in month *t*; *S*_*t *_is the seasonal effect in month *t*; and *ε*_*t *_is the Gaussian random error.

**Figure 4:**
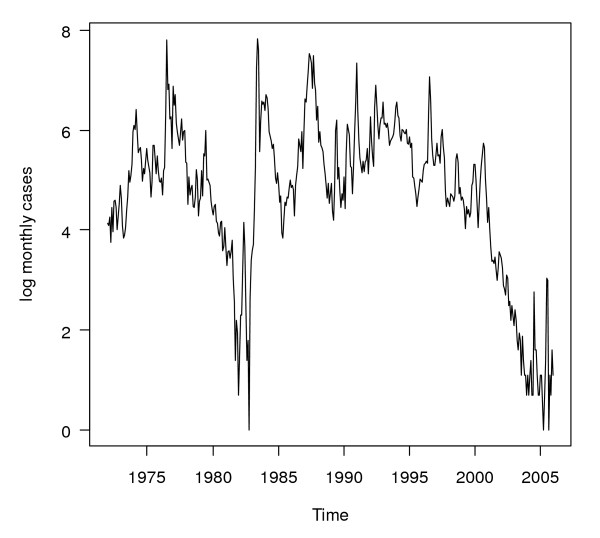
**Logarithmically transformed monthly malaria case counts for Gampaha District**. Logarithmically transformed monthly malaria case counts (after adding the value 1 to all data) for Gampaha District.

As an example, Figure 4 shows the logarithmically transformed series for Gampaha district. The long term fluctuations *m*_*t *_in the logarithmically transformed monthly district malaria case count series were calculated using a 13-point centred smoothing filter with the months at the extremes given half weight:

*m*_*t *_= ^1^/_12_(0.5 *y*'_*t*-6 _+ *y*'_*t*-5 _+ ... + *y*'_*t *_+ ... + *y*'_*t*+5 _+ 0.5 *y*'_*t*+6_)/12.

**Figure 5:**
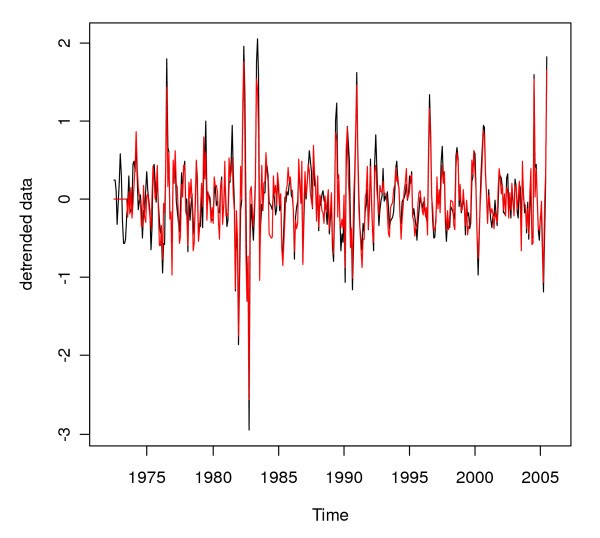
**Detrended (pre-whitened) logarithmically transformed monthly malaria case counts for Gampaha District**. Detrended logarithmically transformed monthly malaria case counts (black line) and pre-whitened detrended logarithmically transformed monthly malaria case counts (red line) for Gampaha District.

Smoothing was performed using the function "decompose" of the package "stats" in the software R [16]. From the detrended series *ζ*_*t *_= *y*'_*t *_- *m*_*t *_(Figure 2 and Figure 5). implicitly long term trends caused by population growth were removed. Cross-correlation analysis was applied between the detrended log transformed malaria case time series and untransformed rainfall time series *x*_*t*_. The cross-correlation was estimated for malaria with a lag *l *of zero to twelve months behind rainfall as

$$r_{l}=\sum_{t=1}^{N}\left(x_{t}-\bar{x}\right)\left(\zeta_{t+l}-\bar{\zeta }\right)/Ns_{x}s_{\zeta }$$

where *s*_*x*_,* s*_*ζ *_are the sample standard deviations of observations on *x*_*t *_and *ζ*_*t*_, respectively. The analysis was repeated with logarithmically transformed rainfall time series *x*'_*t *_= log(*x*_*t *_+ 1).

The cross-correlation is calculated as the average over all (calendar) months, and possible varying correlation depending on the season is not accounted for, *i.e. *if rainfall has a strong positive effect on malaria in some months, and a strong negative in others, the average detected cross-correlation could be weak.

Even though the above approach may find strong correlations, these may not be very useful for malaria prediction if aberrations from the long term seasonal mean of rainfall are weakly linked to aberrations from the long term seasonal mean of the malaria case series. In addition, the standard cross-correlation assumes observations are independent, whereas in reality the malaria data are temporally correlated.

### Cross-correlation analysis with pre-whitening

Cross-correlation with the seasonality and autocorrelation removed by simple pre-whitening allows for detection of the time lag(s) of rainfall preceding malaria, at which divergences from the long term seasonal pattern in rainfall time series show strongest correlation with such divergences in detrended malaria case count time series, while minimizing effects of spurious correlations caused by autocorrelation in the time series. This method bears some similarity to anomaly analysis, where the cross-correlation of aberrations from the long term seasonal mean of the explanatory variables is correlated with aberrations from the long term seasonal mean of the response variable. The effect of pre-whitening is to reduce unassociated autocorrelation and/or trends within time series prior to computation of their cross-correlation function (It is well established that autocorrelation within time series results can produce spurious cross-correlations [15]). Simple pre-whitening is used when there is a clear unidirectional influence such as between rainfall and malaria. First, an auto-regressive model is fit to the explanatory variable. The pre-whitened explanatory variable consists of the residuals of this fitted model, whereas the pre-whitened outcome variable consists of the residuals of the same model (with the same parameters) applied to the outcome variable. With the inclusion of seasonality in the autoregressive model, the pre-whitening procedure removes seasonality (and autocorrelation) from the explanatory variable time series, and the same amount of seasonality (and autocorrelation) from the outcome variable time series. It is thus possible that additional seasonality (and autocorrelation) remains in the pre-whitened outcome variable time series.

For each district, multiplicative seasonal auto-regressive integrated moving average (SARIMA) models [17] with all possible combinations of parameters p, q, P, Q ∈ {0, 1, 2} and with d, D ∈ {0, 1}, were evaluated using the Akaike's information criterion (AIC) on untransformed and logarithmically transformed monthly rainfall totals in the period from January 1971 to December 2005.

The selected SARIMA model was then used to pre-whiten both the rainfall time series and detrended (smoothed) logarithmically transformed malaria case count time series *ζ*_*t*_. The residuals of both series were used as input for the cross-correlation analysis. The functions "arima" and "ccf" from the R package "stats" were used.

The cross-correlation analyses above have the drawback of masking inter-annual effects of rainfall on malaria time series because of the removal of the strong long term trend fluctuations.

### Inter-annual analysis

In "Inter-annual analysis", the series of differenced logarithmically transformed annual malaria cases was studied to determine if it was correlated to differenced logarithmically transformed total annual rainfall. Unlike the first two approaches, it can not account for the within year effects, but inter-annual effects are not masked.

**Figure 6:**
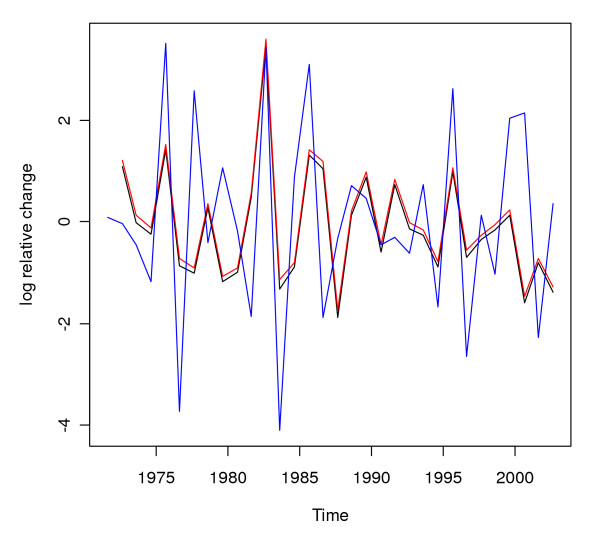
**Differenced logarithmically transformed annual malaria case counts and rainfall for Gampaha District**. Differenced logarithmically transformed annual (the twelve month period starting in April) malaria case counts (black line), malaria case counts with first order auto correlation removed (red line), and the differenced logarithmically transformed annual rainfall with a three month lag shift (the twelve month period starting in January), corrected for autocorrelation in malaria and multiplied by -10 (blue line) for Gampaha District.

**Figure 7:**
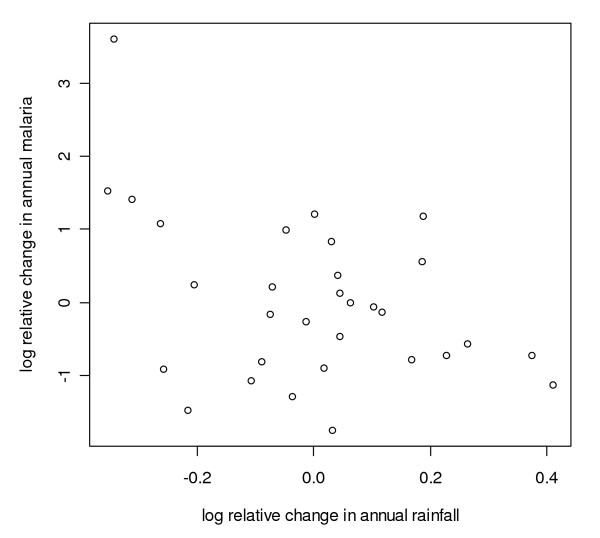
**Scatter plot of differenced logarithmically transformed annual malaria case counts and rainfall for Gampaha District**. Scatter plot of differenced logarithmically transformed annual (the twelve month period starting in April) malaria case counts with first order auto correlation removed against differenced logarithmically transformed annual (the twelve month period starting in January) rainfall corrected for first order auto correlation in malaria for Gampaha District.

The difference Ω_*t*,*k *_= log(*Y*_*t*,*k*_) - log(*Y*_*t*-1,*k*_) = log(*Y*_*t*,*k*_/*Y*_*t*-1,*k*_) reflects the relative change in case numbers between consecutive years [3], where *Y*_*t*,*k *_is the annual malaria case total for year *t*, and the start month *k *of the twelve-month period was either April (*k *= 4) or September (*k *= 9) because seasonally, malaria was lowest in either April or September, depending on the district [13]. Similarly, the relative change in rainfall over 12 month periods preceding the malaria periods with a lag *l *of one to three months was represented by Ξ_*t*,*l*,*k *_= log(*X*_*t*,*k*,*l*_) - log(*X*_*t*-1,*k*,*l*_) = log(*X*_*t*,*k*,*l*_/*X*_*t*-1,*k*,*l*_). Malaria was regressed against rainfall in a first order auto-correlated (AR1) model:

Ω_*t*,*k *_= *φ*_*k*_Ω_*t*-1,*k *_+ *β*_*l*,*k *_(Ξ_*t*,*l*,*k *_- *φ*_*k*_Ξ_*t*-1,*l*,*k*_) + *ε*_*t*,*k*_. The Pearson correlation coefficient between Ω_*t*,*k *_– *φ*_*k*_Ω_*t*-1,*k *_and Ξ_*t*,*k*,*l *_- *φ*_*k*_Ξ_*t*-1,*k*,*l *_was then calculated. Figure 6 and Figure 7 provide an illustration for Gampaha District. The robustness of all significant (*p *≤ 0.1) correlations detected was tested as follows: For each observation it was calculated whether it was influential in terms of dfbeta, dffits, covariance ratios, Cook's distances and the diagonal elements of the hat matrix. Observations which were influential with respect to any of these measures were omitted (one at a time) from the data and the correlation coefficient was recalculated. The weakest correlation among these was recorded.

### Seasonal inter-annual analysis

The effect of rainfall on malaria may depend on the season; therefore, it was of interest to assess the inter-annual relationship between malaria and rainfall for each calendar month in the year. The inter-annual analysis above was modified by replacing Ω_*t*,*k *_with *ω*_*t*,*k*_, and Ξ_*t*,*k*,*l *_with *ξ*_*t*,*k*,*l*_. Here, *ω*_*t*,*k *_represents the average logarithmically transformed malaria count over three months (*e.g. *January – March) differenced with the average logarithmically transformed malaria in the previous twelve months: $\omega_{t,k}=\frac{1}{3}\sum_{k-1}^{k+1}\log\left(y_{t,k}+1\right)-\frac{1}{12}\sum_{k-13}^{k-2}\log\left(y_{t,k}+1\right)$ with *y*_*t*,*k *_the malaria count in month *k *(varied between January and December) and in year *t*. Similarly, $\xi_{t,k,l}=\frac{1}{3}\sum_{k-1-l}^{k+1-l}\log\left(x_{t,k,l}+1\right)-\frac{1}{12}\sum_{k-13-l}^{k-2-l}\log\left(x_{t,k,l}+1\right)$. The seasonally varying correlation coefficients between rainfall and malaria *r*_*k*,*l *_were transformed into *z*_*k*,*l *_values using the Fisher transformation $z_{k,l}=\frac{1}{2}\log\left(\frac{1+r_{k,l}}{1-r_{k,l}}\right)$ and correlated to a three month centred moving average of logarithmically transformed geometric mean seasonal rainfall (similar as depicted in Figure 1, but logarithmically transformed) and its derivative (expressing the change in seasonal rainfall per month).

## Results

### Cross-correlation analysis

**Figure 8:**
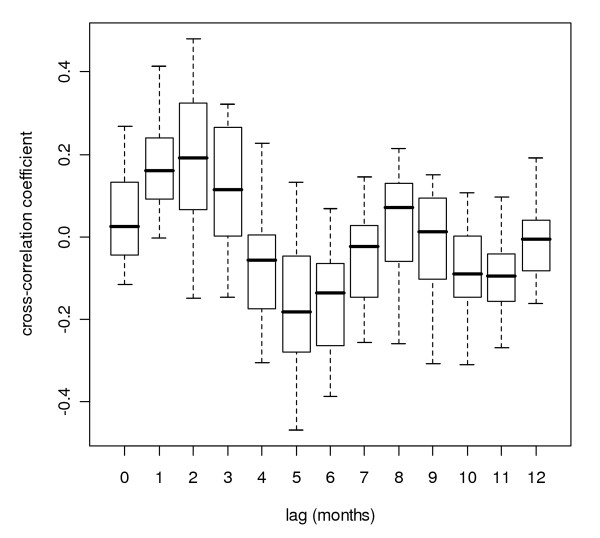
**Cross-correlation box plot**. Box plot of Pearson product-moment correlation coefficients of time series of logarithmically transformed monthly rainfall and (detrended) monthly logarithmically transformed malaria case time series at several lags for districts in Sri Lanka, grouped by lag distance.

**Figure 9:**
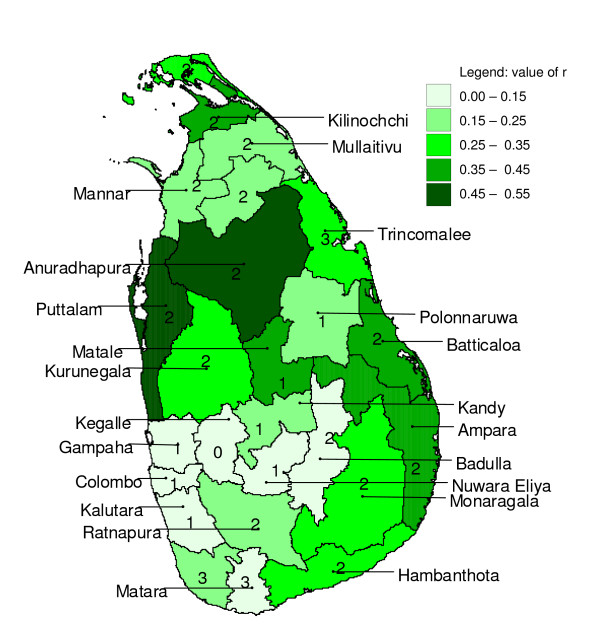
**Mapped maximum cross-correlation coefficients**. Mapped maximum cross-correlation coefficients for logarithmically transformed monthly rainfall preceding (detrended) logarithmically transformed monthly malaria case time series with zero to twelve months for districts in Sri Lanka. Numbers indicate the lag (in months) for which the maximum occurred.

For all districts, a local maximum cross-correlation between malaria and untransformed rainfall or logarithmically-transformed rainfall was found when rainfall was preceding malaria by zero to three months, depending on the district. For 13 out of 25 districts, logarithmic transformation of rainfall improved the cross-correlation (Figure 8), and for some districts the logarithmic transformation of rainfall caused the lag of the local peak correlation to shift by a month. For most districts, the optimum was found at a lag of two months. Neighbouring districts showed similar cross-correlation coefficients at similar lags (Figure 9). The peak correlation coefficient was as high as 0.5 for some districts (*e.g. *Anuradhapura and Puttalam), but very low and not significant for others (*e.g. *Badulla). A local minimum cross-correlation between malaria and untransformed rainfall or logarithmically transformed rainfall was found when rainfall was preceding malaria by four to ten months, depending on the district. For most districts, a second local maximum was found when rainfall was preceding malaria by seven to nine months.

### Cross-correlation analysis with pre-whitening

For pre-whitening, the SARIMA models applied to the (logarithmically transformed) rainfall data showed that for all districts, the model with the lowest AIC had a seasonal component (P = 0, D = 1, Q = 1), and results were very similar among all non seasonal components (p, d, q) tested, except for the components (p = 0, d = 1, q = 0), (p = 1, d = 1, q = 0), and (p = 2, d = 1, q = 0), which gave worse results. The model SARIMA(p = 1, d = 0, q = 0, P = 0, D = 1, Q = 1) was selected.

Figure 5 shows the effect of pre-whitening on the malaria time series for Gampaha district.

**Figure 10:**
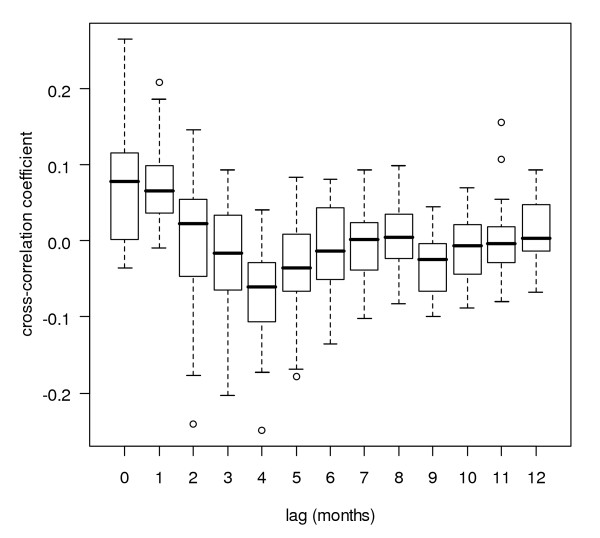
**Cross-correlation box plot after pre-whitening (rainfall log-transformed)**. Box plot of Pearson product-moment correlation coefficients of pre-whitened series of logarithmically transformed monthly rainfall and (detrended) monthly malaria case time series at several lags for districts in Sri Lanka, grouped by lag distance.

**Figure 11:**
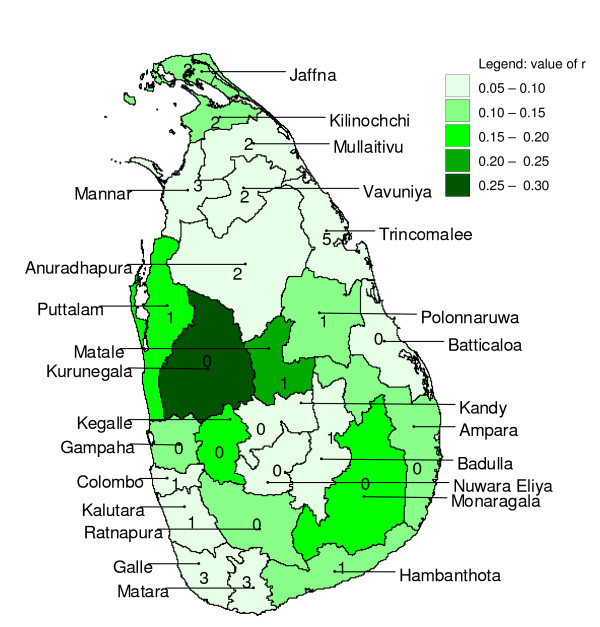
**Mapped maximum cross-correlation coefficients after pre-whitening**. Mapped maximum cross-correlation coefficients for logarithmically transformed rainfall preceding (detrended) logarithmically transformed malaria case time series with zero to twelve months for districts in Sri Lanka, after pre-whitening. Numbers indicate the lag (in months) for which the maximum occurred.

**Figure 12:**
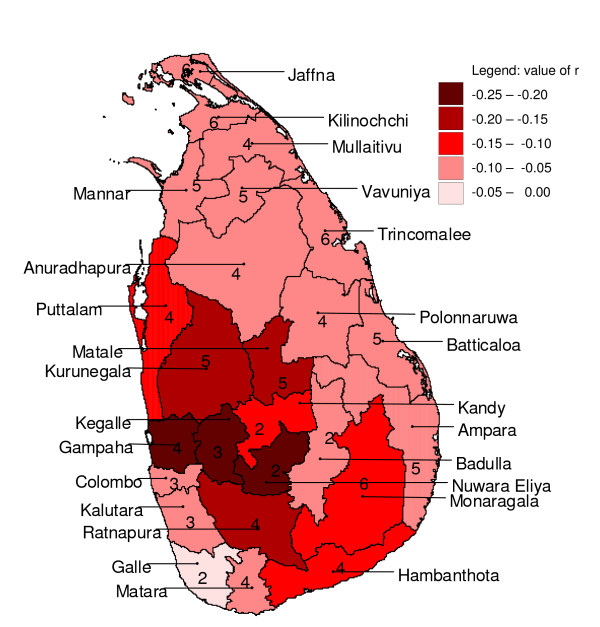
**Mapped minimum cross-correlation coefficients after pre-whitening**. Mapped minimum cross-correlation coefficients for logarithmically transformed monthly rainfall preceding (detrended) logarithmically transformed monthly malaria case time series with zero to twelve months for districts in Sri Lanka, after pre-whitening. Numbers indicate the lag (in months) for which the maximum occurred.

With pre-whitened time series, the cross-correlograms looked entirely different (Figure 10) from the cross-correlograms without pre-whitening (Figure 8). Correlations were generally weaker with pre-whitening than without. Like with the analysis without pre-whitening, neighbouring districts showed similar cross-correlation coefficients at similar lags (Figure 11 and Figure 12). For 18 out of 25 districts, logarithmic transformation of rainfall improved the cross-correlation for the first local maximum, and for 12 out of 25 districts, it improved for the local minimum following the first maximum, as compared to untransformed rainfall. The pre-whitened series showed strongest positive correlations at lags of zero and one month, and only for five out of 25 districts (Puttalam, Kurunegala, Matale, Kegalle and Moneragala, all neighbouring districts except Moneragala) the correlation coefficient was over 0.15. Strongest negative associations were found at lags of two to five months, and only for six out of 25 districts (Gampaha, Kegalle, Kurunegala, Matale, Nuwara Eliya and Ratnapura, all adjoining districts) the correlation coefficient was below -0.15.

### Inter-annual analysis

**Figure 13:**
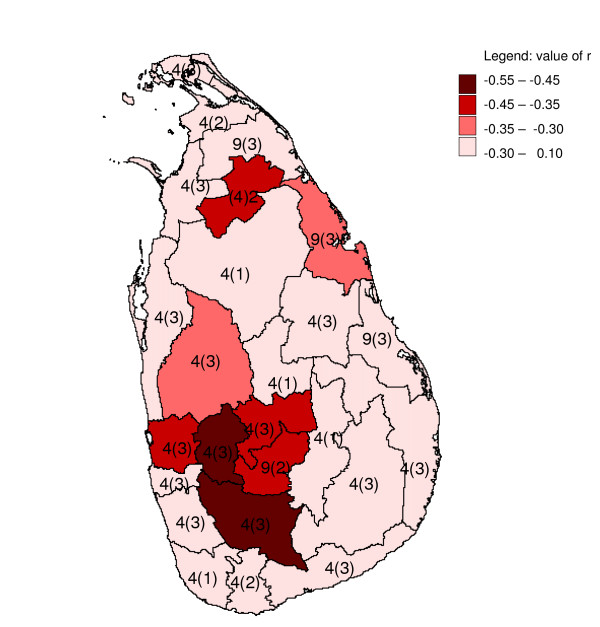
**Mapped minimum inter-annual cross-correlation coefficients**. Mapped minimum cross-correlation coefficients for logarithmically transformed annual rainfall preceding differenced logarithmically transformed annual malaria case time series (with first order auto correlation removed (see methods)), with one to three months for districts in Sri Lanka. Numbers indicate the starting month of the year (4 = April, 9 = November) and between brackets the lag (in months) for which the minimum occurred.

None of the districts showed significant (*p *≤ 0.1, n = 32) positive correlation coefficients (Table 1), and eight districts showed significant negative correlation coefficients (Figure 13). After omitting the influential observation that contributed most to the correlation, for four adjoining districts the correlation coefficients were still significant (*p *≤ 0.1, n = 31). These districts were Kurunegala (r = -0.32, *p *= 0.08), Kegalle (r = -0.48, *p *= 0.007), Kandy (r = -0.34, *p *= 0.063), and Ratnapura (r = -0.52, *p *= 0.003).

**Table 1:** Maximum and minimum Pearson product-moment cross-correlation coefficients, starting month and lag (number of months that malaria case time series are lagged behind) for which the maximum or minimum occurred, and significance of the regression coefficient for logarithmically transformed rainfall and differenced logarithmically transformed annual malaria case time series (n = 32), corrected for first order auto regressive correlation

District	Minimum		Maximum	
	
	cc	start month (lag)	cc	start month (lag)
Ampara	-0.01	4(3)	0.23	9(1)
Anuradhapura	0.08	4(1)	0.27	9(3)
Badulla	-0.09	4(1)	0.30	9(1)
Batticaloa	-0.06	9(3)	0.23	4(1)
Colombo	-0.05	4(3)	0.25	9(3)
Galle	-0.27	4(1)	0.27	9(3)
Gampaha	-0.41*	4(3)	0.03	9(3)
Hambantota	-0.24	4(3)	0.08	9(3)
Jaffna	-0.16	4(3)	0.13	9(1)
Kalutara	-0.10	4(3)	0.25	9(2)
Kandy	-0.40*	4(3)	-0.09	9(3)
Kegalle	-0.55**	4(3)	-0.13	9(3)
Kilinochchi	-0.03	4(2)	0.12	9(1)
Kurunegala	-0.32'	4(3)	0.13	9(1)
Mannar	-0.08	4(3)	0.06	4(1)
Matale	-0.18	4(1)	0.10	9(1)
Matara	-0.11	4(2)	0.13	9(2)
Moneragala	-0.17	4(3)	0.23	9(1)
Mullaitivu	-0.20	9(3)	0.06	4(3)
Nuwara Eliya	-0.36*	9(2)	-0.23	9(3)
Polonnaruwa	-0.22	4(3)	0.17	9(1)
Puttalam	-0.14	4(3)	0.18	9(1)
Ratnapura	-0.52**	4(3)	-0.03	9(1)
Trincomalee	-0.30'	9(3)	-0.07	4(3)
Vavuniya	-0.35'	(4)2	-0.26	(4)1

cc = Pearson product moment correlation coefficient, significance of regression coefficient different from zero: ' = P < 0.10,* = P < 0.05, ** = P < 0.01

### Seasonal inter-annual analysis

**Figure 14:**
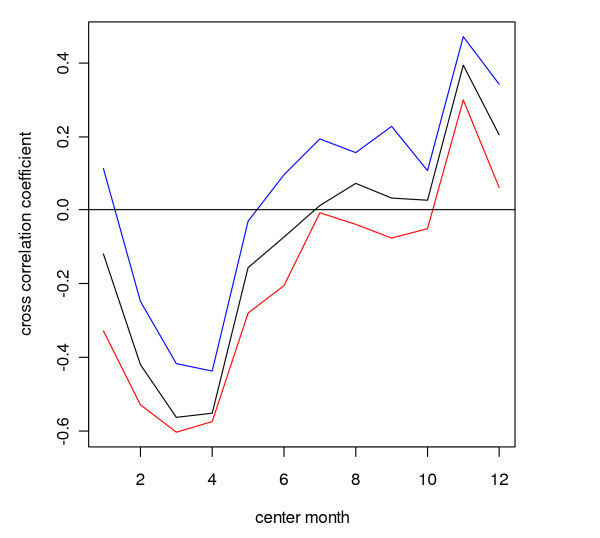
**Cross-correlation coefficients for each rainfall month with malaria lagging one to three months behind for the district of Gampaha**. Cross-correlation coefficients for logarithmically transformed three-monthly rainfall (differenced with the logarithmically transformed rainfall in the preceding twelve months) with logarithmically transformed three-monthly number of malaria cases (differenced with the logarithmically transformed number of malaria in the preceding twelve months), after removing first order auto correlation (see methods)), with the malaria series lagging one (blue line), two (black line) and three (red line) months behind the rainfall series, for the districts of Gampaha in Sri Lanka. The time scale on the horizontal axis reflects the centre month for three rainfall months.

**Figure 15:**
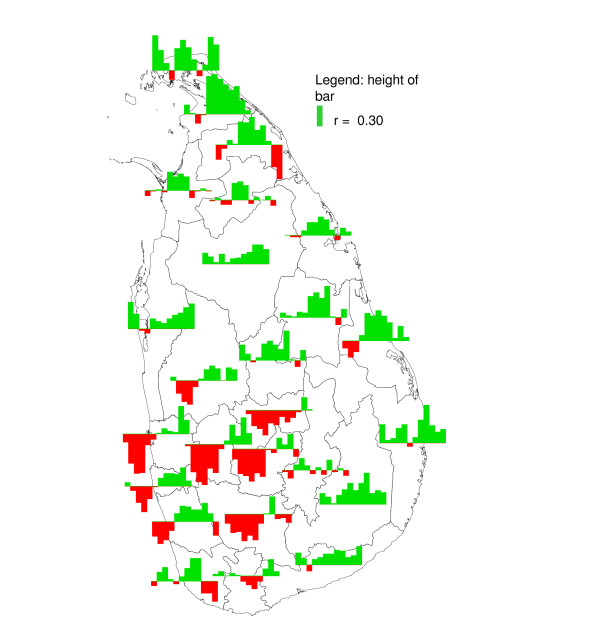
**Mapped seasonal cross-correlation coefficients for malaria lagging two months behind rainfall**. Mapped cross-correlation coefficients for logarithmically transformed three-monthly rainfall (differenced with the logarithmically transformed rainfall in the preceding twelve months) with logarithmically transformed three-monthly number of malaria cases (differenced with the logarithmically transformed number of malaria in the preceding twelve months), after removing first order auto correlation (see methods)), with the malaria series lagging two months behind the rainfall series, for districts in Sri Lanka. The bar on the far left represents January as the centre month of a three months rainfall period; the bar on the far right represents December. Red bars represent negative correlation, blue bars represent positive correlation.

**Figure 16:**
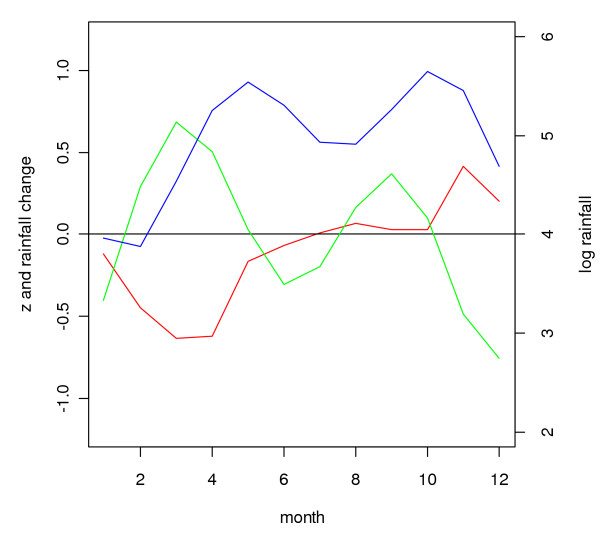
**Correlation coefficients and rainfall for Gampaha District**. Three month centred moving average of logarithmically transformed geometric mean monthly rainfall (in mm per month, calculated over the period January 1971 to December 2005) (blue line on right vertical axis), its derivative representing logarithmically transformed rainfall change per month (green line on left vertical axis) and the Fisher transformed correlation coefficient (red line on left vertical axis) between malaria and rainfall at a lag of two months, found in seasonal inter-annual analysis (see methods) for Gampaha District.

**Figure 17:**
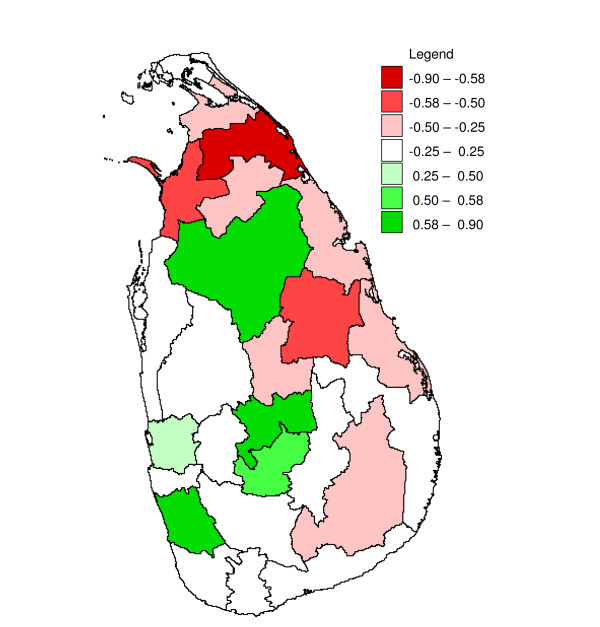
**Correlation between correlation coefficients and rainfall for districts in Sri Lanka**. Mapped correlation coefficient between the Fisher transformed correlation coefficient between malaria and rainfall found in seasonal inter-annual analysis at a lag of two months, (see methods) and a three month centred moving average of logarithmically transformed geometric mean monthly rainfall (in mm per month, calculated over the period January 1971 to December 2005) for districts in Sri Lanka

**Figure 18:**
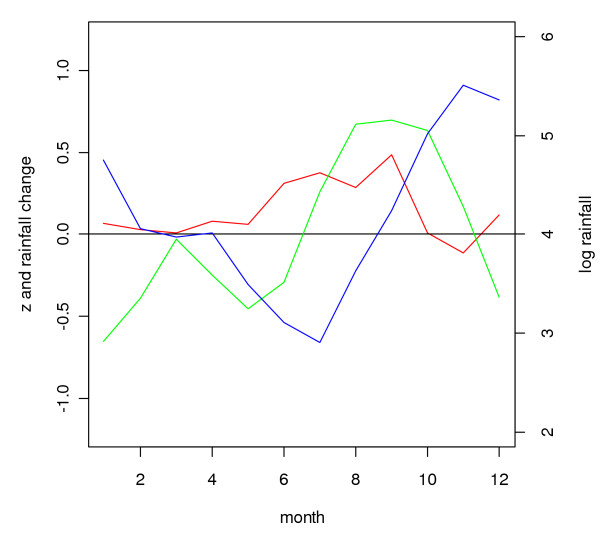
**Correlation coefficients and rainfall for Polonnaruwa District**. Three month centred moving average of logarithmically transformed geometric mean monthly rainfall (originally in mm per month, calculated over the period January 1971 to December 2005) (blue line on right vertical axis), its derivative representing change in logarithmically transformed rainfall per month (green line on left vertical axis) and the Fisher transformed correlation coefficient (red line on left vertical axis) between malaria and rainfall at a lag of two months, found in seasonal inter-annual analysis (see methods) for Polonnaruwa District.

**Figure 19:**
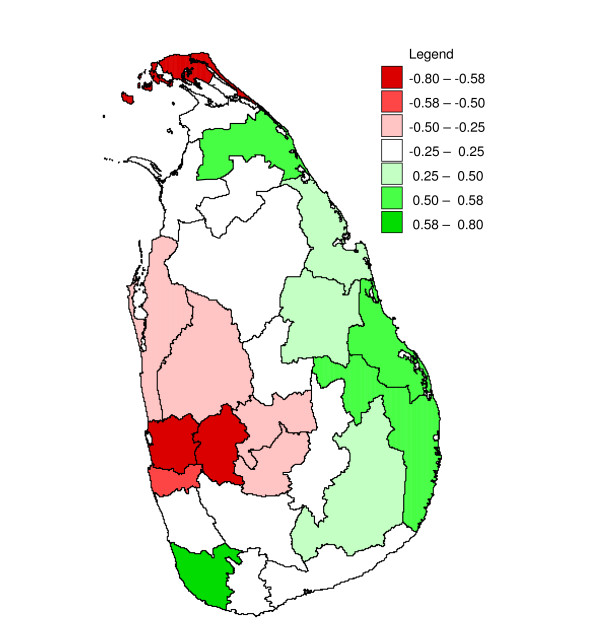
**Correlation between correlation coefficients and change in rainfall for districts in Sri Lanka**. Mapped correlation coefficient between the Fisher transformed correlation coefficient between malaria and rainfall found in seasonal inter-annual analysis at a lag of two months, (see methods) and monthly change in a three month centred moving average of logarithmically transformed geometric mean monthly rainfall (originally in mm per month, calculated over the period January 1971 to December 2005) for districts in Sri Lanka

In a given district, rainfall over a particular three month period (*e.g. *January – March), relative to rainfall in the preceding twelve month period, had in general a similar effect on the malaria count over three months, relative to the malaria in the preceding twelve month period, for malaria following rainfall with a time lag of one (*e.g. *malaria in February – April) to three (*e.g. *malaria in May – June) months, although cross-correlations were stronger positive at a lag of one month and stronger negative at a lag if three months. This is illustrated for the district of Gampaha in Figure 14. The cross-correlation coefficient for rainfall preceding malaria with a lag of two months is presented in Figure 15. This figure shows strong negative correlation coefficients for districts in the centre west of the country for rainfall during February – June. After omitting the influential observation that contributed most to the correlation, the troughs were significant (*p *≤ 0.1, n = 31) for the districts Gampaha, Kegalle and Nuwara Eliya. For Puttalam, Gampaha and Kegalle, significant peaks were observed at the end of the year. In the north and east, some districts showed positive correlation during the middle of the year (significant peaks for the districts Jaffna, Kilinochchi, Batticaloa, Ampara and Moneragala). Jaffna District showed significant positive peaks at the end of the year, whereas for close-by Mullaitivu District the relationship was negative. The Fisher transformed seasonal inter-annual correlation coefficients at a lag of two months were significantly (*p *≤ 0.1, n = 12) negatively correlated to seasonal rainfall in some districts in the east (Mullaitivu, Mannar and Polonnaruwa, Figure 16 and Figure 17), whereas there was a positive correlation to for the districts Anuradhapura, Kandy, Nuwara Eliya and Kalutara. A more smooth picture was obtained by correlating the derivative of seasonal rainfall to the Fisher transformed seasonal inter-annual correlation coefficients. Districts in the centre-west (Gampaha, Kegalle and Colombo) and in the north (Jaffna) showed significant negative correlations (Figure 18 and Figure 19). Districts in the east showed positive correlation (significant for Mullaitivu, Batticaloa and Ampara). Galle also showed a significant positive correlation, but these results should be interpreted with the knowledge that the few infections recorded there are presumed to have been acquired elsewhere.

## Discussion

### Cross-correlation analysis

In some districts in Sri Lanka, malaria case time series and rainfall showed high cross-correlations at short lags as well as at longer lags. While a causal relationship is biologically plausible at a lag of two to four months, it is increasingly less so at longer lag times. Amerasinghe and colleagues [18] found a lag period of 1.5 months between a peak in abundance of *An. culicifacies *immature forms and a peak in malaria cases, in a village in Anuradhapura District. An additional time lag between rainfall and its effect on breeding conditions, depending on conditions such as soil moisture content, has to be included for the calculation of the rainfall – malaria time lag. For most districts, a positive cross-correlation was observed between malaria and rainfall at a lag of two months, confirming the visual impression obtained by studying Figure 1 and Figure 2. It appears, however, that a large part of the detected cross-correlation is due to auto-correlation and concurring cyclical trends, as the cross-correlation analysis with the pre-whitened series (discussed below) showed much smaller cross-correlations at short lags and absence of cross-correlation at longer lags.

### Cross-correlation analysis with pre-whitening

For a few districts, (weak) positive cross-correlations were found in pre-whitened series with no lag (Kegalle, Kurunegala and Moneragala) and at a lag of one month (Matale and Puttalam). With a lag of one month, short term prediction with a one month horizon would be possible. However, a one month lag seems the absolute minimum for the biological pathway from creating suitable breeding conditions to mosquito development, parasite development in the mosquito, the onset of disease symptoms, and eventually the taking of a blood sample. Nevertheless, in a study in China, log transformed malaria and rainfall showed a maximum (positive) effect for malaria lagging one month behind rainfall, when entered into a regression model together with minimum temperature and fixed quarterly effects for seasonality [19]. Cross-correlations of rainfall contemporary with malaria (at a lag of zero months) are of no value for malaria prediction systems because the total monthly rainfall for the future month needs to be known, unless rainfall can be predicted with high certainty. The (strongest) negative cross-correlations, albeit weak, found in the six adjoining districts at lags of two to five months in the centre-west, are in line with other studies that showed that this region (except for Nuwara Eliya district which is situated at high altitude) is particularly prone to epidemics when monsoon rains fail [6]. It is difficult to find explanations for the differences in lag time at which the maximum or minimum cross-correlation occurs among (often neighbouring) districts. Factors that could contribute to these differences are saturation levels and water retention of top soils, factors related to differences in malaria endemicity, and differences in temperature (mainly caused by differences in altitude). However, given the generally weak cross-correlations, a large part of the inter-district variation in time-lag of maximum or minimum cross-correlation could have been caused by stochastic noise.

After pre-whitening, the cross-correlations found were very weak. Only if rainfall can explain that part of the variation in a malaria time series that cannot be explained by autocorrelation and repetition of seasonal patterns, a rainfall covariate could contribute to a malaria forecasting system. It was only for two out of the six districts (Gampaha and Ratnapura) with strongest negative correlation (situated in the centre west) that Briët and colleagues [10] found some contribution of rainfall to malaria prediction in seasonal ARIMA models at a lag of two months, and they found no improvement for the districts Matale and Puttalam at a lag of one month.

### Inter-annual analysis

Some studies in neighbouring India [20,21], with comparable total annual rainfall and strong seasonality in rainfall, have tried but failed to find a significant correlation between annual rainfall and malaria. These studies did not consider differencing or detrending the data. A study in Ingwavuma and Ubombo districts in KwaZulu-Natal province in South Africa, with less annual rainfall than Sri Lanka, also failed to find such correlation between annual malaria and rainfall time series, but it did find significant positive correlations between the difference of successive twelve-monthly (July to June, corresponding to the local malaria season) logarithmically transformed malaria case totals and summer (November – March) rainfall (and temperature) [3], while the long term trends were attributed to non-climatic factors [22]. Likewise, a study in Botswana, also with less annual rainfall than Sri Lanka, found a positive correlation between (detrended) annual malaria anomalies and December – February rainfall [23]. In the present study, strong negative correlations were found between differenced annual malaria and rainfall for a contiguous group of districts in the centre-west (with high annual rainfall), and these results were somewhat in line with the results found in the cross-correlation analysis with pre-whitening. This area in particular has been repeatedly affected by malaria epidemics during droughts in the pre-malaria control era [6], and apparently malaria control has not changed this dynamic. Although initially significant negative correlations were detected for the drier districts Vavuniya and Trincomalee, the correlations in these districts were not very robust to influential observations. The data quality in the north-east has been compromised by the armed conflict in the region, and for some districts (particularly Vavuniya) some missing data were imputed. The strong (negative) inter-annual correlations found for the districts in the centre-west provides hope for the development of long term malaria forecasting systems involving long term weather forecasts, provided these systems have sufficient capabilities to predict rainfall anomalies up to a year in advance, which is currently not feasible. It is tempting to attribute the inverse direction of the relationship between rainfall and malaria found in this analysis as compared to the direction found in Southern Africa to the difference in annual rainfall, although other important differences exist, notably in malaria vector species.

### Seasonal inter-annual analysis

The results of the seasonal inter-annual analysis supported the theory that rainfall varies in its effect on malaria transmission depending on the season. These effects may cancel out when averaged over the entire calendar year (inherent to the first three approaches studied), and therefore, it seems that malaria forecasting systems incorporating rainfall need to take this seasonally varying effect into account. Note, however, that Briët and colleagues [10] found limited improvement in malaria prediction with a seasonally varying rainfall effect for only three districts.

There was a marked difference in the season-varying effect of rainfall on malaria between the south-western quadrant of the country and the rest of the country. In the south west, the effect was strongly negative during February – June, whereas in the other quadrants, often a positive effect was found during April – September. In most districts (except in the north-eastern quadrant), also a (weak) positive effect was found in December or January.

Similar to the explanation of the spatial variation in malaria endemicity by spatial variation in annual rainfall, the spatial variation in the (seasonally varying) effect of rainfall on malaria may be explained by spatial variation in (seasonal) rainfall. In the south west, rainfall is normally lowest between November – April, in contrast with the rest of the country, where the April – September trough is (much) deeper in the rainfall climatology (Figure 1). The classic biological explanation for the epidemics in the centre-west of the country is that failure of the south-west monsoon (that normally occurs between February – July, affecting mostly the wet and intermediate zones in the west) will cause the already low rivers (relative to the rest of the year) to stagnate and create breeding sites for *An. culicifacies*. Thus the strong negative correlation found for rainfall occurring in February to May/June, especially during the first half of the first rainfall season, could be explained. During the second half of the first rainfall season, when rivers flow, the negative effect is negligible. However, the positive correlation at the end of the year, occurring just after the peak of the second rainfall season, is in contrast with this reasoning. Possibly different breeding sites play a role at that time of the year. In the north and east, the climate is particularly dry from February – September. Here, rainfall during the middle of the year will provide the water required for mosquito breeding and humidity for survival, explaining the positive relationship found during the middle of the year, after the driest months. During the north-east monsoon (October – January) the rivers flow normally abundantly, and a negative correlation might be expected (based on the observations in the centre west of the country) during this period, but only for one district with poor quality malaria data (Mullaitivu) this was apparent, whereas for another close by district (Jaffna), the opposite was observed. There is no evidence that this mechanism plays a role in the north and east. Furthermore, in the north and east, the highest malaria peak is normally observed in January (just after the peak in the north-east monsoon rains). This is in line with the fact that early malariologists considered rivers not to be intimately involved in the mechanism of epidemic malaria in the dry zone [6]. The results from the analysis of correlation between Fisher transformed correlation coefficients and rainfall also suggested a different mechanism in the centre west from that in the rest of the country.

The fact that positive correlations were stronger at a lag of one month, and negative correlations were stronger at a longer lag of three months may be explained as follows: Within a one month period, rainfall can influence malaria transmission and cases positively by providing humidity which increases mosquito survival. One month might not be long enough for rainfall to influence malaria cases through an effect on mosquito breeding. A negative effect of rainfall on mosquito breeding (for instance less than normal rainfall which might cause river pooling, which will have a delay in itself) will need a longer lag period to translate into a change in malaria cases.

### Limitations of this study

This study was limited to linear rainfall – malaria relationships. For a better understanding of the biological mechanisms behind the observed relationships between rainfall and malaria cases, the link between rainfall and mosquito breeding and survival should be included. Long, high quality time series of entomological data were unfortunately not available for this purpose. Rainfall influenced variables, such as soil moisture saturation and river flow, are more directly linked to specific malaria vector breeding conditions. However, such variables are more expensive to measure and therefore often estimated using rainfall, offering little advantages unless for instance human interference with river flow, for purposes such as irrigation or power generation, could be taken into account. Such interference disrupts the relationship between rainfall and river flow, and hence the relationship between rainfall and malaria [24]. Apart from rainfall and rainfall related variables, another variable that is expected to have a strong temporal effect on malaria case count time series is malaria control intervention. This variable was not taken into account due to incomplete data. Also, control methods and insecticides have changed over time, making it a complex covariate. Temperature was not studied as it was considered of less importance than rainfall, showing little temporal variability (because Sri Lanka is situated close to the equator), and a large part of its temporal variability being governed by rainfall. Except for the hill country, situated in the centre of Sri Lanka, the temperature is conducive to malaria transmission throughout the year. Other environmental factors that are often considered in malaria studies are altitude and land use. These were not taken into account as these do not fluctuate (strongly) over time. Another limitation is the use of Gaussian models on transformed count data, whereas negative binomially distributed methods on untransformed data (personal communication) may have been more appropriate. This study was performed on aggregated cases of *Plasmodium falciparum *and *Plasmodium vivax*. Although the seasonality of *P. vivax*. is slightly less marked than that of *P. falciparum*, possibly caused by relapses of *P. vivax *occurring well after infection, the seasonality is very similar [13]. In the current study, it was presumed that cases were infected in the district where they were recorded. In the large spatial units of districts only a small percentage of cases may have been acquired elsewhere, and these would mostly be expected to have been acquired in neighbouring districts with similar rainfall patterns. Nevertheless, in districts with normally very low transmission such as Galle, Nuwara Eliya and Colombo, the proportion of cases from elsewhere might be much higher, and the relationships between rainfall and malaria for these districts should be interpreted with care.

## Conclusion

Although malaria and rainfall showed high cross-correlations in many districts in Sri Lanka, variation from normal monthly malaria counts patterns showed limited cross-correlation with variation from normal monthly rainfall patterns, and therefore rainfall may have limited use for predicting malaria. Seasonally varying effects of rainfall on malaria case counts may explain weak cross-correlations in pre-whitened series (as the cross-correlation analysis did not allow for a seasonally varying effect). There was a marked difference in the seasonally varying effect between the south-western quadrant and the rest of the country, which was probably related to differences in rainfall, but also to spatially different water requirements for optimum breeding conditions for the main malaria vector in Sri Lanka.

## Authors' contributions

OJTB conceptualized and conceived of the analysis, performed the data treatment and analysis, and drafted the manuscript. PV participated in the conceptualization, edited the manuscript and critically revised the statistical methodology. DMG participated in the conceptualization of the study and edited the manuscript. GNLG provided the data and helped define the scope of the paper. PHA participated in defining the approach to analysis, edited and critically reviewed the paper for intellectual content. All authors read and approved of the manuscript.

## References

[B1] Teklehaimanot HD, Lipsitch M, Teklehaimanot A, Schwartz J (2004). Weather-based prediction of Plasmodium falciparum malaria in epidemic-prone regions of Ethiopia I. Patterns of lagged weather effects reflect biological mechanisms. Malar J.

[B2] Worrall E, Connor SJ, Thomson MC (2007). A model to simulate the impact of timing, coverage and transmission intensity on the effectiveness of indoor residual spraying (IRS) for malaria control. Trop Med Int Health.

[B3] Craig MH, Kleinschmidt I, Nawn JB, Le Sueur D, Sharp BL (2004). Exploring 30 years of malaria case data in KwaZulu-Natal, South Africa: part I. The impact of climatic factors. Trop Med Int Health.

[B4] Clemesha WW (1934). Brief account of the natural history of malaria in Ceylon. Ceylon J Sci.

[B5] Gill CA (1936). Some points in the epidemiology of malaria arising out of the study of the malaria epidemic in Ceylon in 1934-35. Trans R Soc Trop Med Hyg.

[B6] Rustomjee KJ (1944). Observations upon the epidemiology of malaria in Ceylon.

[B7] Mendis C, Gamage-Mendis AC, De Zoysa AP, Abhayawardena TA, Carter R, Herath PR, Mendis KN (1990). Characteristics of malaria transmission in Kataragama, Sri Lanka: a focus for immuno-epidemiological studies. Am J Trop Med Hyg.

[B8] Van der Hoek W, Konradsen F, Perera D, Amerasinghe PH, Amerasinghe FP (1997). Correlation between rainfall and malaria in the dry zone of Sri Lanka. Ann Trop Med Parasitol.

[B9] De Alwis R, Wijesundere A, Ramasamy MS, Ramasamy R, Ramasamy R (1990). Epidemiology of malaria in Aralaganvila in the Polonnaruwa district. Current status of malaria research in Sri Lanka.

[B10] Briët OJT, Vounatsou P, Gunawardena DM, Galappaththy GNL, Amerasinghe PH (2008). Models for short term malaria prediction in Sri Lanka. Malar J.

[B11] Thomson MC, Connor SJ (2001). A framework for field research in Africa:  Malaria early warning systems: concepts, indicators and partners.

[B12] Konradsen F, Amerasinghe FP, Van der Hoek W, Amerasinghe PH (2000). Malaria in Sri Lanka: Current knowledge on transmission and control.

[B13] Briët OJT, Gunawardena DM, Van der Hoek W, Amerasinghe FP (2003). Sri Lanka malaria maps. Malar J.

[B14] Briët OJT, Vounatsou P, Amerasinghe PH (2008). Malaria seasonality and rainfall seasonality in Sri Lanka are correlated in space. Geospatial Health.

[B15] Chatfield C (2004). The analysis of time series: an introduction.

[B16] (2007). R. http://www.r-project.org.

[B17] Box GEP, Jenkins GM (1968). Some recent advances in forecasting and control. Appl Statist.

[B18] Amerasinghe PH, Amerasinghe FP, Konradsen F, Fonseka KT, Wirtz RA (1999). Malaria vectors in a traditional dry zone village in Sri Lanka. Am J Trop Med Hyg.

[B19] Bi P, Tong S, Donald K, Parton KA, Ni J (2003). Climatic variables and transmission of malaria: a 12-year data analysis in Shuchen County, China. Public Health Rep.

[B20] Dev V, Dash AP (2007). Rainfall and malaria transmission in north-eastern India. Ann Trop Med Parasitol.

[B21] Singh N, Sharma VP (2002). Patterns of rainfall and malaria in Madhya Pradesh, central India. Ann Trop Med Parasitol.

[B22] Craig MH, Kleinschmidt I, Le Sueur D, Sharp BL (2004). Exploring 30 years of malaria case data in KwaZulu-Natal, South Africa: part II. The impact of non-climatic factors. Trop Med Int Health.

[B23] Thomson MC, Mason SJ, Phindela T, Connor SJ (2005). Use of rainfall and sea surface temperature monitoring for malaria early warning in Botswana. Am J Trop Med Hyg.

[B24] Wijesundera MS (1988). Malaria outbreaks in new foci in Sri Lanka. Parasitol Today.

